# Self-Reported Efficacy of Cannabis and Other Complementary Medicine Modalities by Parkinson's Disease Patients in Colorado

**DOI:** 10.1155/2015/874849

**Published:** 2015-03-02

**Authors:** Taylor Andrew Finseth, Jessica Louise Hedeman, Robert Preston Brown, Kristina I. Johnson, Matthew Sean Binder, Benzi M. Kluger

**Affiliations:** ^1^Deptartment of Neurology, University of Colorado Anschutz Medical Campus, Aurora, CO 80045, USA; ^2^Department of Economics, University of Colorado Denver, Denver, CO 80217, USA; ^3^Colorado School of Public Health, University of Northern Colorado, Greeley, CO 80639, USA

## Abstract

*Introduction*. Complementary and alternative medicine (CAM) is frequently used by Parkinson's disease (PD) patients. We sought to provide information on CAM use and efficacy in PD patients in the Denver metro area with particular attention to cannabis use given its recent change in legal status. *Methods*. Self-administered surveys on CAM use and efficacy were completed by PD patients identified in clinics and support groups across the Denver metro area between 2012 and 2013. *Results*. 207 patients (age 69 ± 11; 60% male) completed the survey. Responses to individual CAM therapy items showed that 85% of respondents used at least one form of CAM. The most frequently reported CAMs were vitamins (66%), prayer (59%), massage (45%), and relaxation (32%). Self-reported improvement related to the use of CAM was highest for massage, art therapy, music therapy, and cannabis. While only 4.3% of our survey responders reported use of cannabis, it ranked among the most effective CAM therapies. *Conclusions*. Overall, our cross-sectional study was notable for a high rate of CAM utilization amongst PD patients and high rates of self-reported efficacy across most CAM modalities. Cannabis was rarely used in our population but users reported high efficacy, mainly for nonmotor symptoms.

## 1. Introduction

Complementary and alternative medicine (CAM) is defined by the National Center of Complementary and Alternative Medicine as a group of diverse medical and healthcare systems, practices, and products that are not considered an integral part of conventional medicine [1]. CAM modalities include alternative medical systems like traditional Chinese medicine; bioactive substances like vitamins or herbal supplements; manipulative therapies like massage or chiropractic; mind-body therapies like yoga or relaxation; and energy therapies like Reiki. CAM is estimated to be used by approximately 38% of the US population with an estimated 34 billion dollars spent on CAM products and services annually [2, 3].

Parkinson's disease (PD) is the second most common neurodegenerative disorder following Alzheimer's disease and leads to impairments in function and quality of life through both motor and nonmotor symptoms. A recent review article examining the use of CAM in PD found that the usage rates and most commonly used modalities by country were as follows: South Korea (76%, oriental medicine), USA (40%, vitamins), UK (39%, massage), Sweden (34%, acupuncture), and Argentina (26%, acupuncture) [4].

The last study of CAM use by PD patients in the USA was conducted in 2001 on outpatients of a tertiary academic practice in Baltimore [5]. Our study sought to provide updated figures on the prevalence and spectrum of CAM use in PD patients in Colorado across multiple providers and to provide data on self-reported efficacy of CAM for various symptoms.

In addition, we specifically investigated usage of cannabis amongst the PD population in Colorado. Medical cannabis has been legally available with authorization from a physician in Colorado since 2000 and available to patients in retail outlets with a prescription since 2010. Recreational use of cannabis without a prescription was legalized in Colorado in November 2012, just prior to the start of our study. There have been few clinical trials of cannabis in PD, most of which were underpowered with uncontrolled series suggesting benefit for both motor and nonmotor symptoms and controlled trials with negative or inconclusive results [6]. In contrast, a single survey based study from the Czech Republic reported high rates of use (25%) and benefit for multiple symptoms (>40%) [7]. Although this came from a large sample (*N* = 339) there was a 54% nonresponse rate raising issues of reporting bias. Studies of medical cannabis in the general population show frequent use for pain, insomnia, and anxiety/depression, all of which are common nonmotor symptoms of PD [8]. Due to the paucity of literature on the subject and potential interest and benefit in our PD population, we sought to gather information on cannabis usage and self-reported efficacy amongst the Colorado PD population.

## 2. Methods

### 2.1. Standard Protocol Approvals, Registrations, and Patient Consents

The Colorado Multiple Institutional Review Board approved this study and, given that it was an anonymous survey not collecting identifying information, waived consent requirements.

### 2.2. Patient Surveys

A self-administered survey was provided to patients at the University of Colorado Hospital Movement Disorders Clinics and PD support groups in the Denver metro area between November 2012 and August 2013. The anonymous survey was completed either in person or at the patient's convenience and returned to the study coordinator in person or via mail.

The survey asked patients to indicate past or current use of individual CAM modalities provided in a list format for management of symptoms associated with Parkinson's disease (see Supplementary Material for complete survey available online at http://dx.doi.org/10.1155/2015/874849). If they had used a modality, they were asked to answer follow-up questions of whether they had noticed “great improvement,” “some improvement,” “no improvement,” or “worsening/side effects” with that modality. They were also asked if the modalities used had been associated with improvement in any of the following areas: quality of life, mood, sleep, energy, or motor symptoms. The survey collected self-reported demographic information, disease characteristics. Data on multivitamins, diet, exercise, and physical therapy were also collected for comparison purpose. Exercise and physical therapy were not considered CAM as they are considered standard of care for PD [9]. Multivitamins and diet were not considered CAM for purposes of estimating CAM prevalence because they are commonly employed by the general public for general health maintenance. We did not ask about the specific use of diet for PD or use of specific diets (e.g., gluten-free).

Incomplete surveys were not discarded for purposes of analysis. Completion rates for the majority of items ranged from 95 to 97% with the exception of income, which had a 74% completion rate. Three of the 210 completed surveys were discarded due to suspected inaccuracy of demographic information: one with age listed as 2006 and two surveys with initial diagnosis at ages 18 and 26. We did not track refusal rate, but we estimate it was below 10% and typically due to time constraints.

### 2.3. Statistical Analysis

Study data were input and managed using Research Electronic Data Capture (REDCap) hosted at the University of Colorado Denver Anschutz Medical Campus [10]. Statistical analyses were performed using SAS 9.3 (Cary, NC) and included unadjusted descriptive statistics. Determination of associations between demographic and clinical variables and CAM use was performed using Student's *t*-test for continuous variables and Chi-square test or Fisher's exact test (if cell frequencies were less than 5) for categorical variables. *P* < 0.05 was considered statistically significant.

## 3. Results

### 3.1. Demographics

Two hundred and seven patients with PD completed the survey between November 2012 and August 2013.  Table 1 describes the demographics of our cohort. 82% had at least some college education and 33% had graduate level education. A majority of the patients were of higher socioeconomic status with 60% reporting income above $60,000. Average duration of Parkinson's symptoms was 8.15 (±6.9) years.

### 3.2. Estimated Prevalence and Self-Reported Efficacy of CAM Use

52% of patients reported past or current use of CAM therapies for treatment of PD symptoms. However, when surveys were analyzed based on specific CAM therapy items, 85% of patients demonstrated current or recent use of at least one form of CAM (Table 2). 88% of responders reported that their physician was aware of their CAM use. Vitamins, not including multivitamins, were the most frequently used CAM (66%), of which vitamin E and CoQ10 were the most commonly used. Other common forms of CAM included prayer (49%), massage (45%), and relaxation techniques (32%).

We further analyzed self-reported efficacy of various CAMs as well as several commonly accepted nonpharmacological therapies such as physical therapy and exercise (Table 3). Self-reported improvement with CAM modalities varied from 29% to 86% and was similar to well accepted non-CAM therapies. Most therapies had a response rate (defined by report of some or great improvement due to the therapy) of 60% or greater. Massage and meditation had the highest response rates of the commonly used therapies. Music therapy, cannabis, and chiropractic also had high rates of reported effectiveness, although in a smaller proportion of the total sample. The CAM therapies with the highest rate of reported improvement in specific domains are listed in Table 3. Acupuncture, exercise, homeopathy, and spiritual therapy each had one respondent report worsening or side effects. Side effects were not reported with any other therapy.

Nine (4.3%) responders reported use of cannabis to treat their PD symptoms. Of those nine, four had fourteen or more years of education and eight were white. Ages ranged from 49 to 75, and these patients reported PD symptom duration ranging from 2 to 11 years. Seven rated their overall health as good or excellent, while two rated their overall health as fair. Five reported “great improvement” in their symptoms with cannabis. In particular, five reported improvement in their mood and sleep, and two reported improvement in their motor symptoms and quality of life. No one reported worsening of symptoms or side effects.

### 3.3. Association of Demographic/Clinical Variables and CAM Use

There were no associations found between demographic or disease characteristics and overall CAM use. Regarding the five most common forms of CAM, females were significantly more likely to utilize massage, relaxation techniques, and acupuncture and African Americans and Asians were significantly more likely to utilize relaxation techniques. No significant correlations were found for age, education, or income with the five most commonly reported forms of CAM. Disease characteristics of years since diagnosis, age at initial diagnosis, number of PD medications used, and current health status were also examined without significant correlations with the top 5 most common forms of CAM. There were no significant differences in the demographics of patients using cannabis compared to demographics of all survey respondents.

## 4. Discussion

Our study was the first to systematically examine self-reported efficacy of CAM therapies in PD patients including cannabis. We found higher CAM usage rates than prior studies for the general US population (28–54%) and similar or higher rates reported for other neurological disorders like stroke (30%), sciatica (47%), memory loss (47%), migraines (50%), epilepsy (70%), pediatric neurology (78%), and multiple sclerosis (81%) [11–15]. In addition, our results suggest a higher percentage of PD patients use CAM than that found by Rajendran et al., who estimated 40% CAM use amongst a similar cohort of PD patients in Maryland [5]. Due to the large discrepancy between the findings of these two studies, it is worthwhile to explore possible explanations. One explanation may be regional differences. The Western United States has been shown to have higher CAM usage rates amongst the general population compared to other regions [16, 17]. Both Denver and Boulder have a very visible complementary and alternative medicine community, which may lead to increased awareness and access to CAM. The two studies also used different techniques; Rajendran et al. interviewed consecutive patients of a single practitioner with low refusal rate. Our study, in contrast, studied patients from multiple practitioners at both academic centers and community support groups, data was self-reported anonymously, and refusal rate was higher. This design is more prone to overestimate the true prevalence of CAM use given possibility of self-selection bias. Despite this possibility, we believe the true prevalence of CAM use amongst our population is likely higher than that found by Rajendran et al. and may reflect regional variability as well as societal trends of increasing CAM use in the past decade [5]. Given this suspected regional variability, our study's findings may not accurately reflect CAM usage patterns amongst PD patients on a national level.

The relative use of the various CAM modalities in the USA is similar to that found by Rajendran et al. in 2001. Vitamins and herbal supplements remain the most utilized, followed by prayer, massage, relaxation, and acupuncture. Our findings also demonstrate differences in CAM use by PD patients in Colorado when compared to PD patients in other countries [4]. While acupuncture is the most commonly used CAM therapy for PD worldwide, it is just the fifth most popular CAM in our cohort which may be reflective of low insurance coverage of acupuncture services in the USA. In contrast, vitamin usage in the USA greatly exceeds that in any other region studied to date [18–20]. The reasons for high rates of vitamin usage in the USA may be explained by their wide availability and ease of use. We also suspect that part of the discrepancy between self-reported rates of CAM use and calculated rates may be due to patients not considering vitamins as a form of CAM. Despite their popularity, vitamins had low reported efficacy in improvement in quality of life or other metrics compared to other CAM modalities. Notably, the two most commonly used vitamins (vitamin E and CoQ10) have both been shown in large randomized clinical trials to not improve PD outcomes [21, 22].

Self-reported efficacy for many CAM therapies was high for both motor and nonmotor symptoms. Reported improvement in certain domains suggests areas where further clinical research may be useful. Meditation, art therapy, and cannabis all had responder rates of greater than 50% for mood improvement and, with the exception of cannabis, might be considered to augment standard pharmacotherapy or psychotherapeutic approaches. In the author's experience, cannabis is potentially helpful for anxiety but not depression and there is in fact evidence for cannabis use increasing depressive symptoms [23]. Cannabis had the highest responder rate for sleep improvement at 55% and might be considered for refractory insomnia not responsive to first-line agents. Motor improvement rates approaching 50% were reported by patients using massage, chiropractic, and tai chi. Notably, there is strong evidence from randomized clinical trials to support tai chi use and consistent but less rigorous data for massage [24–26]. Chiropractic has not been studied and caution may be warranted for manipulations, particularly involving the cervical spine [27].

The use of cannabis as a therapeutic agent for various medical conditions has been well documented, but there are few studies looking specifically at its use for treating PD symptoms [6, 28–31]. While only a small number of participants in our study reported use of cannabis for PD, those that did reported benefits in mood (56%), sleep (56%), motor symptoms (22%), and quality of life (22%). Our results differ from those found in a survey of Czech PD patients from 2004 in several important ways [28]. Usage rates are lower (4.3% versus 25.1%) and motor response was lower (22% versus 44.7%). However, the few randomized clinical trials performed to date have failed to substantiate any objective benefits and suggest side effects including lightheadedness and drowsiness are fairly common [32]. Our data suggests that PD patients in our cohort tend to utilize cannabis for its impact on nonmotor symptoms rather than motor symptoms, and it was rated as the most effective therapy for sleep and mood improvement amongst all CAMs. There is a single small observational study also suggesting that cannabis therapies may be helpful for psychosis in PD patients with dementia [33]. While the role of cannabis in PD is not yet clearly delineated we suspect its use will grow in popularity given the recent legalization in select states, the wide range of potential formulations in these states, and increased media attention. Potential side effects of cognitive impairment, apathy, fatigue, or balance have not been fully examined and will be important to consider when planning any controlled trials of medical cannabis.

Prior studies have shown that age of onset of PD and high income and higher education levels are correlated with CAM use. Our study did not confirm these correlations, perhaps due to the very high rate of CAM use amongst the entire population. Our study also found that 88% of participants reported their physician was aware of their CAM therapy. This is in contrast to previous reports of less than half of PD patients reporting CAM use to their physician. It is difficult to draw conclusions on the reason for this difference but this may reflect cultural shifts in the openness and awareness of physicians regarding CAM as well as shifts in patients' perceptions.

Our study has several notable limitations. Refusal rate was not tracked and therefore our estimate of CAM prevalence in the general PD population may be biased. Presumably, patients that do not use CAM would be more likely to refuse the survey and could lead to an overestimation of CAM usage. In addition, despite our efforts to reach out to the community, the demographics of our study population were typical of a tertiary medical center population, with predominantly white men and highly educated patients, and therefore likely do not fully represent the PD population as a whole. Furthermore, patients living in Colorado may be more likely to utilize CAM than in other regions. All data was self-reported and the survey was self-administered in an anonymous fashion, allowing for misinterpretation of survey questions and making confirmation of responses impossible. There is also a risk that some patients who completed the survey through PD support groups may not have had PD although we assume that this would be a rare occurrence. While we asked patients only to indicate their use of CAM if specifically used for PD, responders may have indicated use even if not specifically for PD. It is also likely that patients used CAM for reasons that we did not ask about such as pain. Furthermore, self-reported efficacy is prone to overreporting of improvement and placebo effect and cannot substitute for data from randomized clinical trials.

Considering the continued growth of CAM use in the general population and amongst PD populations, further randomized clinical trials regarding efficacy of CAM would help physicians guide patients in best use of CAM therapy. Future studies should ideally be placebo-controlled given findings of up to 50% positive placebo effects in prior trials in PD patients [34].

## Supplementary Material

Supplementary Material: An electronic copy of the complete survey used in our study is provided here as it was presented to patients.

## Figures and Tables

**Table 1:** Characteristics of the study population.

Age, y, mean (SD)	68.9 (10.9)
Male, *n* (%)	125 (60.4)
Female, *n* (%)	82 (39.6)
Race	
White, *n* (%)	190 (92.68)
African American, *n* (%)	3 (1.46)
Hispanic, *n* (%)	8 (3.90)
Asian/Pacific Islander, *n* (%)	3 (1.46)
Native American, *n* (%)	1 (0.49)
Education level	
Less than high school, *n* (%)	3 (1.47)
High school, *n* (%)	31 (14.98)
College, *n* (%)	102 (49.28)
Graduate school, *n* (%)	68 (32.85)
Income	
<$20,000, *n* (%)	15 (9.8)
$20,000–39,999, *n* (%)	21 (13.72)
$40,000–59,999, *n* (%)	25 (16.33)
$60,000–$99,999, *n* (%)	39 (25.5)
>$100,000, *n* (%)	53 (34.6)
Duration of PD, y, mean (SD)	8.15 (6.9)
Age of initial diagnosis, years, mean (range)	61.2 (32–87)
Young onset cases (≤45 y), *n* (%)	6 (3.0)

**Table 2:** Most frequently used CAM treatments^a^.

Rank	Treatment	Number using treatment [*n* (%)]	Number reporting any improvement [*n* (%)]
1	Vitamins	137 (66%)	52 (38%)
2	Prayer	124 (59%)	83 (67%)
3	Massage	94 (45%)	81 (86%)
4	Relaxation	66 (32%)	46 (70%)
5	Acupuncture	61 (29%)	33 (66%)
6	Meditation	57 (27%)	44 (77%)
7	Yoga	55 (26%)	40 (73%)
8	Herbal	52 (25%)	21 (40%)
9	Chiropractor	44 (21%)	34 (77%)
10	Music	44 (21%)	36 (82%)
11	Imagery	33 (16%)	25 (76%)
12	Tai chi	25 (12%)	18 (72%)
13	Spiritual therapy	24 (11%)	18 (75%)
14	Art therapy	21 (10%)	18 (86%)
15	Energy therapy	19 (9%)	10 (53%)
16	Reflexology	14 (7%)	6 (43%)
17	Homeopathy	14 (7%)	4 (29%)
18	Biofeedback	13 (6%)	6 (46%)
19	Fava beans	11 (5%)	5 (45%)
20	Cannabis	9 (4%)	7 (78%)
21	Hypnosis	7 (3%)	2 (29%)
22	Qi gong	6 (3%)	2 (33%)
23	Ayurveda	6 (3%)	2 (33%)

	Exercise	187 (89%)	159 (85%)
	Physical therapy (PT)	114 (55%)	94 (82%)
	Multivitamin (MVI)	116 (56%)	38 (33%)
	Cognitive behavioral therapy (CBT)	20 (10%)	18 (90%)

^a^Exercise, PT, MVI, and CBT are not considered CAM but are included for comparison purposes.

**Table 3:** Most efficacious CAM treatments^a^.

Rank	Any improvement (%)	QOL improvement (%)	Mood improvement (%)	Sleep improvement (%)	Energy improvement (%)	Motor improvement (%)
1	Massage (86%)	Spiritual (54%)	Med (60%)	Cannabis (55%)	Yoga (35%)	Massage (46%)
2	Art (86%)	Art (52%)	Art (57%)	Massage (31%)	Med (35%)	Chiro (45%)
3	Music (82%)	Chiro (48%)	Cannabis (55%)	Relax (26%)	Acu (30%)	Music (36%)
4	Cannabis (78%)	Massage (47%)	Music (45%)	Med (25%)	Massage (30%)	Tai chi (36%)
5	Med (77%)	Med (44%)	Spiritual (42%)	Acu (18%)	Music (30%)	Yoga (35%)

	Exercise (85%)	Exercise (52%)	Exercise (41%)	Exercise (35%)	Exercise (43%)	Exercise (40%)
	PT (82%)	PT (42%)	PT (25%)	PT (17%)	PT (32%)	PT (44%)
	MVI (33%)	MVI (20%)	MVI (8%)	MVI (3%)	MVI (22%)	MVI (3%)
	CBT (90%)	CBT (45%)	CBT (45%)	CBT (20%)	CBT (10%)	CBT (25%)

PT: physical therapy, MVI: multivitamin, CBT: cognitive behavioral therapy, Chiro: chiropractic, Med: meditation, Acu: acupuncture, and Relax: relaxation techniques.

^a^Exercise, PT, MVI, and CBT are not considered CAM but are included for comparison purposes.
