# Supplementary material for: Self-Reported Efficacy of Cannabis and Other Complementary Medicine Modalities by Parkinson's Disease Patients in Colorado
Source: Evid Based Complement Alternat Med. 2015 Mar 2;2015:874849. doi: 10.1155/2015/874849 (PMC4363882; doi:10.1155/2015/874849)
Supplement: Supplementary file 1 — Supplementary Material: An electronic copy of the complete survey used in our study is provided here as it was presented to patients. [file 874849.f1.pdf]

Post Card Consent

Approved
NOV 02 2012
COMIRB

Date: **Valid for Use Through:**

Study Title: Complementary and Alternative Medicine (CAM) usage and Parkinson's disease

Principal Investigator: Kristina Johnson

COMIRB No: 12-1375
Version Date:
Version No:

You are being asked to be in this research study because you can give feedback on the types of Complementary and Alternative Medicine (CAM) therapies that Parkinson's disease patients are using to manage symptoms associated with Parkinson's disease.

If you join the study, you will be asked to fill out a survey both before and after the presentation.

This study is designed to learn more about the types of CAM therapies that Parkinson's disease patients are using to manage Parkinson's disease symptoms. Future Parkinson's disease and CAM therapy studies will be based on the information gathered from these surveys.

Every effort will be made to protect your privacy and confidentiality. No identifiers will be used in the survey – i.e. no names, date of birth or other identifying information will be collected in this survey.

You have a choice about being in this study. You do not have to be in this study if you do not want to be.

If you have questions, you can call *Kristina Johnson* at 303-905-2531. You can call and ask questions at any time.

You may have questions about your rights as someone in this study. If you have questions, you can call the COMIRB (the responsible Institutional Review Board). Their number is (303) 724-1055.

Complementary and Alternative Medicine Survey

Approved
NOV 02 2012
COMIRB

Date ______________________

Survey ID ______________________

Please complete the following information.

1. Sex/Gender
   - ☐ Male
   - ☐ Female

2. Race
   - ☐ White
   - ☐ African-American
   - ☐ Hispanic
   - ☐ Asian/Pacific Islander
   - ☐ Native American

Please indicate the following in the space provided.

3. Number of years of education (e.g. 12 years = high school graduate, 16 years = college graduate, etc.) ______________________

4. Household income ______________________

5. Age at initial diagnosis of Parkinson's disease ______________________

6. Number of years of Parkinson's disease symptoms ______________________

7. How many medications are you using currently to treat Parkinson's disease symptoms? ______________________

8. Please rate your current overall health
   - ☐ Poor
   - ☐ Fair
   - ☐ Good
   - ☐ Very good
   - ☐ Excellent

9. Have you used or do you currently use Complementary and Alternative Medicine (CAM) therapies to treat Parkinson's disease (PD) symptoms?
   - ☐ Yes
   - ☐ No

10. If you use CAM therapies, is your physician aware that you use these therapies?
    - ☐ Yes
    - ☐ No
    - ☐ Yes for some therapies, and No for some therapies
    - ☐ N/A

11. If you share some CAM therapies with your physician, but not others, please indicate the CAM therapies that your physician does NOT know about. ______________________

How often do you use the following CAM therapies?

Natural Products

	Never	Daily	Weekly	Monthly	Yearly
Vitamin E	☐	☐	☐	☐	☐
Co-Q10	☐	☐	☐	☐	☐
Fava beans	☐	☐	☐	☐	☐
Mucuna	☐	☐	☐	☐	☐
Multi-vitamin	☐	☐	☐	☐	☐
Other vitamins/nutritional supplements	☐	☐	☐	☐	☐
Other herbal supplements	☐	☐	☐	☐	☐
Medical marijuana	☐	☐	☐	☐	☐

Mind/Body Medicine

	Never	Daily	Weekly	Monthly	Yearly
Acupuncture and acupressure	☐	☐	☐	☐	☐
Tai Chi	☐	☐	☐	☐	☐
Meditation/mindfulness	☐	☐	☐	☐	☐
Yoga	☐	☐	☐	☐	☐
Hypnotherapy	☐	☐	☐	☐	☐
Qi Gong	☐	☐	☐	☐	☐
Relaxation techniques	☐	☐	☐	☐	☐
Imagery/visualization	☐	☐	☐	☐	☐
Biofeedback	☐	☐	☐	☐	☐

Manipulative and Body-Based Practice

	Never	Daily	Weekly	Monthly	Yearly
Massage therapy	☐	☐	☐	☐	☐
Chiropractic techniques	☐	☐	☐	☐	☐
Reflexology	☐	☐	☐	☐	☐
Physical therapy	☐	☐	☐	☐	☐

Whole Medical Systems

	Never	Daily	Weekly	Monthly	Yearly
Aruveda	☐	☐	☐	☐	☐
TCM - Traditional Chinese Medicine	☐	☐	☐	☐	☐

Other Therapies

	Never	Daily	Weekly	Monthly	Yearly
Diet	☐	☐	☐	☐	☐
Exercise	☐	☐	☐	☐	☐
Prayer	☐	☐	☐	☐	☐
Energy healing (Reiki, Healing Touch)	☐	☐	☐	☐	☐
Homeopathy	☐	☐	☐	☐	☐
Spiritual healing	☐	☐	☐	☐	☐
Cognitive behavioral therapy	☐	☐	☐	☐	☐
Music therapy	☐	☐	☐	☐	☐
Art therapy	☐	☐	☐	☐	☐
Chelation	☐	☐	☐	☐	☐
Colonic hydrotherapy	☐	☐	☐	☐	☐
IV therapies	☐	☐	☐	☐	☐
Ionic foot baths	☐	☐	☐	☐	☐

Additional therapies

	Never	Daily	Weekly	Monthly	Yearly
Other: ______________	☐	☐	☐	☐	☐
Other: ______________	☐	☐	☐	☐	☐
Other: ______________	☐	☐	☐	☐	☐
Other ______________	☐	☐	☐	☐	☐

If you use CAM therapies for PD, have you noticed improvement in your symptoms, and if so how much?

Natural Products

	N/A	Greatly improved	Some improvement	No improvement	Worsening or side effects
Vitamin E	☐	☐	☐	☐	☐
Co-Q10	☐	☐	☐	☐	☐
Fava beans	☐	☐	☐	☐	☐
Mucuna	☐	☐	☐	☐	☐
Multi-vitamin	☐	☐	☐	☐	☐
Other vitamins/nutritional supplements	☐	☐	☐	☐	☐
Other herbal supplements	☐	☐	☐	☐	☐
Medical marijuana	☐	☐	☐	☐	☐

Mind/Body Medicine

	N/A	Greatly improved	Some improvement	No improvement	Worsening or side effects
Acupuncture or acupressure	☐	☐	☐	☐	☐
Tai Chi	☐	☐	☐	☐	☐
Meditation/mindfulness	☐	☐	☐	☐	☐
Yoga	☐	☐	☐	☐	☐
Hypnotherapy	☐	☐	☐	☐	☐
Qi Gong	☐	☐	☐	☐	☐
Relaxation techniques	☐	☐	☐	☐	☐
Imagery/visualization	☐	☐	☐	☐	☐
Biofeedback	☐	☐	☐	☐	☐

Manipulative and Body-Based Practice

	N/A	Greatly improved	Some improvement	No improvement	Worsening or side effects
Massage therapy	☐	☐	☐	☐	☐
Chiropractic techniques	☐	☐	☐	☐	☐
Reflexology	☐	☐	☐	☐	☐
Physical therapy	☐	☐	☐	☐	☐

Whole Medical Systems

	N/A	Greatly improved	Some improvement	No improvement	Worsening or side effects
Aruveda	☐	☐	☐	☐	☐
TCM - Traditional Chinese Medicine	☐	☐	☐	☐	☐

Other Therapies

	N/A	Greatly improved	Some improvement	No improvement	Worsening or side effects
Diet	☐	☐	☐	☐	☐
Exercise	☐	☐	☐	☐	☐
Prayer	☐	☐	☐	☐	☐
Energy healing (Reiki, Healing Touch)	☐	☐	☐	☐	☐
Homeopathy	☐	☐	☐	☐	☐
Spiritual healing	☐	☐	☐	☐	☐
Cognitive behavioral therapy	☐	☐	☐	☐	☐
Music therapy	☐	☐	☐	☐	☐
Art therapy	☐	☐	☐	☐	☐
Chelation	☐	☐	☐	☐	☐
Colonic hydrotherapy	☐	☐	☐	☐	☐
IV therapies	☐	☐	☐	☐	☐
Ionic foot baths	☐	☐	☐	☐	☐

Additional therapies

	N/A	Greatly improved	Some improvement	No improvement	Worsening or side effects
Other: ______________	☐	☐	☐	☐	☐
Other: ______________	☐	☐	☐	☐	☐
Other: ______________	☐	☐	☐	☐	☐
Other: ______________	☐	☐	☐	☐	☐

If you use CAM therapies for PD, which of the following have improved? (Mark all that apply.)

Natural Products

	Quality of life	Mood	Sleep	Energy	Motor symptoms (e.g. tremor, slowness, stiffness)	Other
Vitamin E	☐	☐	☐	☐	☐	☐
CO-Q10	☐	☐	☐	☐	☐	☐
Fava Beans	☐	☐	☐	☐	☐	☐
Mucuna	☐	☐	☐	☐	☐	☐
Multi-vitamin	☐	☐	☐	☐	☐	☐
Other vitamins/nutritional supplements	☐	☐	☐	☐	☐	☐
Other herbal supplements	☐	☐	☐	☐	☐	☐
Medical marijuana	☐	☐	☐	☐	☐	☐

Mind/Body Medicine

	Quality of life	Mood	Sleep	Energy	Motor symptoms (e.g. tremor, slowness, stiffness)	Other
Acupuncture or acupressure	☐	☐	☐	☐	☐	☐
Tai Chi	☐	☐	☐	☐	☐	☐
Meditation/mindfulness	☐	☐	☐	☐	☐	☐
Yoga	☐	☐	☐	☐	☐	☐
Hypnotherapy	☐	☐	☐	☐	☐	☐
Qi Gong	☐	☐	☐	☐	☐	☐
Relaxation techniques	☐	☐	☐	☐	☐	☐
Imagery/visualization	☐	☐	☐	☐	☐	☐
Biofeedback	☐	☐	☐	☐	☐	☐

Manipulative and Body-Based Practice

	Quality of life	Mood	Sleep	Energy	Motor symptoms (e.g. tremor, slowness, stiffness)	Other
Massage therapy	☐	☐	☐	☐	☐	☐
Chiropractic techniques	☐	☐	☐	☐	☐	☐
Reflexology	☐	☐	☐	☐	☐	☐
Physical therapy	☐	☐	☐	☐	☐	☐

Whole Medical Systems

	Quality of life	Mood	Sleep	Energy	Motor symptoms (e.g. tremor, slowness, stiffness)	Other
Aruveda	☐	☐	☐	☐	☐	☐
TCM - Traditional Chinese Medicine	☐	☐	☐	☐	☐	☐

Other Therapies

	Quality of life	Mood	Sleep	Energy	Motor symptoms (e.g. tremor, slowness, stiffness)	Other
Diet	☐	☐	☐	☐	☐	☐
Exercise	☐	☐	☐	☐	☐	☐
Prayer	☐	☐	☐	☐	☐	☐
Energy healing (Reiki, Healing Touch)	☐	☐	☐	☐	☐	☐
Homeopathy	☐	☐	☐	☐	☐	☐
Spiritual healing	☐	☐	☐	☐	☐	☐
Cognitive behavioral therapy	☐	☐	☐	☐	☐	☐
Music therapy	☐	☐	☐	☐	☐	☐
Art therapy	☐	☐	☐	☐	☐	☐
Chelation	☐	☐	☐	☐	☐	☐
Colonic hydrotherapy	☐	☐	☐	☐	☐	☐
IV therapies	☐	☐	☐	☐	☐	☐
Ionic foot baths	☐	☐	☐	☐	☐	☐

Additional therapies not previously mentioned

	Quality of life	Mood	Sleep	Energy	Motor symptoms (e.g. tremor, slowness, stiffness)	Other
Other: ______________	☐	☐	☐	☐	☐	☐
Other: ______________	☐	☐	☐	☐	☐	☐
Other: ______________	☐	☐	☐	☐	☐	☐
Other: ______________	☐	☐	☐	☐	☐	☐

12. If you checked other in any category, please specify. ______________________
